# Retrospective analysis of the efficacy and safety of anlotinib plus sintilimab (anti-PD-1) as maintenance therapy in advanced pediatric solid tumors

**DOI:** 10.3389/fonc.2025.1518987

**Published:** 2025-09-10

**Authors:** Rui Zhao, Luyang Chang, Chengyi Zhang, Rongheng He, Xudong Wei

**Affiliations:** ^1^ Department of Hematology, Henan Institute of Hematology, The Affiliated Cancer Hospital of Zhengzhou University & Henan Cancer Hospital, Zhengzhou, Zhengzhou, China; ^2^ Department of Translational Research and Cellular Therapeutics, Beckman Research Institute, City of Hope, Duarte, CA, United States; ^3^ Central Laboratory, The Affiliated Cancer Hospital of Zhengzhou University & Henan Cancer Hospital, Zhengzhou, China

**Keywords:** advanced pediatric tumors, anlotinib, sintilimab, maintenance therapy, efficacy, safety

## Abstract

**Background:**

Advanced solid tumors in children have limited maintenance treatment options. This study assessed the effectiveness and safety of anlotinib in conjunction with sintilimab as maintenance therapy for advanced pediatric solid tumors in real-world settings.

**Methods:**

This single-institution retrospective study was conducted at the Affiliated Cancer Hospital of Zhengzhou University from November 2019 to October 2023. Forty-six patients with advanced pediatric solid tumors who achieved partial response or stable disease following first-line (22/46) or second-line (24/46) chemotherapy subsequently received maintenance therapy with a combination of anlotinib and sintilimab. The primary endpoint was median progression-free survival (mPFS). Secondary endpoints included median overall survival (mOS), disease control rate (DCR), and safety.

**Results:**

After a median follow-up of 21.8 months (95% CI, 16.5–27.1), the mPFS was 25.3 months (95% CI, 7.0–43.6) in the first-line treatment group and 13.3 months (95% CI, 7.2–19.4) in the second-line treatment group. The mOS in the first-line and second-line treatment groups was 38.2 months (95% CI, 22.2–54.1) and 16.5 months (95% CI, 12.6–20.4), respectively. The DCR was 50.0% (11/22; 95% CI, 28–72) in the first-line group and 37.5% (9/24; 95% CI, 19–59) in the second-line group. Most treatment-related adverse events were grade 1–2. The most common grade 3–4 adverse event was anemia (2/46, 4.3%).

**Conclusion:**

These results indicate that maintenance therapy using anlotinib combined with sintilimab could be a safe and effective treatment option for advanced pediatric tumors.

## Introduction

An estimated 1 in 257 children and adolescents will be diagnosed with cancer before the age of 20 (1, 2). According to a report from The Lancet, between 2018 and 2020, an estimated 121,145 cancer cases were diagnosed among children and adolescents in China (3) Although the survival rate of childhood cancer in high-income countries is over 80%, the 5-year survival rate in low- and middle-income countries is less than 30% (4, 5). Many patients with advanced pediatric tumors still face suboptimal outcomes (6, 7). To achieve sustained long-term remission and survival, pediatric tumor patients with metastatic disease at early diagnosis require maintenance treatment after completing initial therapy.

Immune checkpoint inhibitor (ICI) therapy relieves immune suppression of T lymphocytes via immune checkpoints (8). In the past few years ICIs have become a major pillar of treatment for more than 15 types of adult cancer (9). However, the pediatric experience has been notably different, with initial studies demonstrating limited efficacy of ICIs in children (10). These results underscore the differences in immunogenicity between pediatric and adult cancers (11). Compared with adult tumors, the immune microenvironment of childhood tumors exhibits significant heterogeneity. Studies have shown that tumor-infiltrating lymphocytes (TILs) in childhood tumors are predominantly naïve T cells, with lower cytotoxic T lymphocyte (CTL) activity and a relatively higher proportion of regulatory T cells (Treg). This immune cell composition may limit the endogenous antitumor immune response (12). Additionally, the cytokine network in the microenvironment of childhood tumors is imbalanced, such as high expression of immunosuppressive factors like IL-10, which further inhibits T cell activation and proliferation (13).

Clinical trials indicate that nivolumab is well tolerated and shows antitumor efficacy in pediatric populations with relapsed or refractory non-central nervous system (CNS) solid tumors or lymphoma (14). However, when used alone, neither nivolumab nor pembrolizumab showed activity in sporadic pediatric solid tumor histotypes (15). Published reviews have evaluated clinical trials combining PD-1 inhibitors with other therapeutic approaches, providing insights into methodologies for conducting immunotherapy clinical trials (16).

The relationship between the upregulation of angiogenic signaling pathways and tumor immune suppression has been established (17, 18). Vascular endothelial growth factor (VEGF) impairs tumor-antigen presentation by hindering the maturation of dendritic cells and upregulating the expression of programmed death-ligand 1 (PD-L1) on dendritic cells, thereby suppressing the function of CD8+ T cells (19, 20). Anlotinib, a potent tyrosine kinase inhibitor (TKI), demonstrates promising multitarget activity against CKIT, VEGFR, PDGFR, and FGFR pathways (21). *In vitro* studies have confirmed that anlotinib facilitates tumor vessel normalization through CD4+ T cells, significantly inhibits neuroblastoma (NB) cell growth, and effectively prevents systemic immunosuppression (22).

Further studies have confirmed that the combined application of anlotinib and PD-1 checkpoint inhibitors has a clear synergistic mechanism: their combination can effectively reduce the immunosuppression caused by PD-L1 upregulation after monotherapy, significantly prolong the duration of vascular normalization, and ultimately strongly promote the regression of neuroblastoma (23).

Currently, multiple clinical trials on anlotinib combined with PD-1 inhibitors for the treatment of advanced solid tumors are underway. For example, a phase II trial conducted by Liu Kai et al. reported that anlotinib combined with toripalimab (another anti-PD-1 drug) showed a favorable objective response rate (58.3%) in patients with advanced gastric cancer (24).

However, clinical data targeting the pediatric population remain limited. Notably, there have been no previous clinical studies explicitly evaluating the efficacy and safety of the combined use of anlotinib and sintilimab in children with tumors, and there is a particular lack of data on whether their combination for maintenance treatment can benefit children with advanced tumors. This is precisely where the innovation of our study lies—in exploring the application value of this combination regimen in pediatric patients.

This retrospective analysis focuses on the application of anlotinib combined with the PD-1 inhibitor sintilimab in pediatric patients, aiming to systematically evaluate the synergistic efficacy and safety of this combination regimen in the treatment of advanced pediatric solid tumors.

## Methods

### Study design and participants

This single institutional, retrospective study was conducted at the Affiliated Cancer Hospital of Zhengzhou University to evaluate the antitumor activity and safety of anlotinib combined with sintilimab as maintenance treatment for patients who achieved a partial response or stable disease after completing at least four cycles of first-line or second-line chemotherapy. First-line treatment: Refers to the initial systemic therapy administered to patients following a confirmed diagnosis, as per standard clinical guidelines for their specific tumor type (e.g., vincristine-based regimens for rhabdomyosarcoma, cisplatin-based regimens for osteosarcoma). This was the first anticancer treatment received by all patients in the first-line group. Second-line treatment: Defined as the first subsequent systemic therapy administered after disease progression, recurrence, or intolerance to first-line treatment. This included regimens with different mechanisms of action from the first-line therapy (e.g., switching from alkylating agents to tyrosine kinase inhibitors) to ensure therapeutic distinction. Eligible patients were: ① less than 18 years old at the initial onset of cancer; ② initial diagnosis of disease at stage IIIb or IV; ③ at least one evaluable disease that can be accurately measured according to Response Evaluation Criteria in Solid Tumours (RECIST) version 1.1 (25); ④ Eastern Cooperative Oncology Group (ECOG) performance status score of 0–2, expected survival time of over 3 months. The exclusion criteria included: 1) fewer than one cycle of anlotinib combined with sintilimab; 2) incomplete data.

### Ethics statement

The study involving human participants was reviewed, and the protocol was approved by the institutional review board and ethics committee of the Affiliated Cancer Hospital of Zhengzhou University (No. 2021-KY-0214-001). All guardians signed the drug informed consent form before patients received the designated treatment.

### Treatment schedule

Oral anlotinib was given at a dose of 8 mg (<35 kg), 10 mg (≥35 kg), once daily, 2 weeks on/1 week off. Intravenous sintilimab (anti-PD-1) was given as follows: ≥40 kg, 200 mg every 3 weeks; <40 kg, 3 mg/kg every 3 weeks. Maintenance therapy continued until disease was stable for at least 1 year or until disease progression. For patients with grade 3–4 adverse events, anlotinib was temporarily discontinued until the adverse events resolved to grade ≤1 (median duration of discontinuation: 5 days; range, 3–8 days). Treatment was then resumed at a reduced dose (from the initial 12 mg/day to 8 mg/day).

### Assessment of response and toxicity

RECIST 1.1 was used to evaluate the responses to anlotinib combined with sintilimab. Tumor assessment was performed using computed tomography (CT) or magnetic resonance imaging (MRI) within 1 month before treatment initiation (baseline). After treatment initiation, tumor responses were confirmed at least every 2 cycles (6 weeks) thereafter. NCI CTC v5 (National Cancer Institute Common Toxicity Criteria for Adverse Events, version 5.0) was used to grade adverse events of anlotinib combined with sintilimab treatment (26).

### Observation endpoints

mOS was defined as the time from the beginning of induction chemotherapy until death from any cause or the end of the last follow-up. Progression-free survival (PFS) was defined as the duration from the beginning of anlotinib treatment to disease progression or death, whichever occurred first. Disease control rate (DCR) was defined as the proportion of complete response (CR), partial response (PR), and stable disease (SD). The response and toxicity were monitored from the beginning of anlotinib combined with sintilimab treatment to the occurrence of death or end of follow-up (October 11, 2023).

### Statistical methods

All analyses were conducted using SPSS statistical software version 23 (IBM Inc., IL) and Prism version 8.0. mPFS and mOS were evaluated using the Kaplan–Meier method. The Clopper–Pearson method was used to compare DCR between first- and second-line treatment groups, and differences between the curves were assessed using the log-rank test. A p-value of less than 0.05 was considered statistically significant.

## Results

### Patient characteristics

A total of 46 patients were screened between November 13, 2019, and October 11, 2023. The enrolled patients were divided into two groups: 22 (47.8%) received first-line therapy, and 24 (52.2%) received second-line therapy before anlotinib combined with sintilimab maintenance treatment. The baseline characteristics are presented in **Table 1**. The median age at anlotinib combined with sintilimab initiation was 10.25 (0.3–17.5) years; 26 (56.6%) patients were male, and 20 (43.4%) were female. The histopathology types included rhabdomyosarcoma (n = 12), osteosarcoma (n = 10), neuroblastoma (n = 9), Ewing’s sarcoma (n = 5), synovial sarcoma (n = 1), alveolar soft part sarcoma (n = 1), Wilms’ tumor (n = 2), retinoblastoma (n = 3), desmoid-type fibromatosis (n = 1), nasopharyngeal carcinoma (n = 1), and primitive neuroectodermal tumors (n = 1). All patients had an acceptable ECOG performance status score (≤2). Before receiving maintenance therapy, all patients had received different treatments, including surgery, several lines of chemotherapy, radiotherapy, or hematopoietic stem cell transplantation (HSCT). Among all the patients, 6 (13.0%) received radiotherapy and 2 (4.3%) received HSCT in the first-line therapy group, and 3 (6.5%) received radiotherapy and 3 (6.5%) received HSCT in the second-line therapy group.

**Table 1:** Baseline characteristics of the patients.

Characteristics	First-line therapy (n=22, 47.8)	Second-line therapy (n=24, 52.2)	Total (n=46, 100)
Median age (range) years	11.75 (0.3-17.5)	9 (1.3-16)	10.25 (0.3-17.5)
Gender			
Female	11 (23.9)	19 (41.3)	20 (43.5)
Male	11 (23.9)	15 (32.6)	26 (56.5)
ECOG performance status			
0	12 (26.1)	9 (19.6)	21 (45.7)
1	8 (17.4)	10 (21.7)	18 (39.1)
2	2 (4.3)	5 (10.9)	7 (15.2)
Histology			
Rhabdomyosarcoma	5 (10.9)	7 (15.2)	12 (26.1)
Osteosarcoma	4 (8.7)	6 (13.0)	10 (21.7)
Neuroblastoma	4 (8.7)	5 (10.9)	9 (19.6)
Ewing’s sarcoma	2 (4.3)	3 (6.5)	5 (10.9)
Retinoblastoma	1 (2.2)	2 (4.3)	3 (6.5)
Will’s tumor	2 (4.3)	0 (0)	2 (4.3)
Alveolar soft-part sarcoma	1 (2.2)	0 (0)	1 (2.2)
Synovial sarcoma	1 (2.2)	0 (0)	1 (2.2)
Desmoid-type Fibrosarcoma	1 (2.2)	0 (0)	1 (2.2)
Nasopharyngeal carcinoma	1 (2.2)	0 (0)	1 (2.2)
Primitive neuro-ectodermal tumors	0 (0)	1 (2.2)	1 (2.2)
Received radiotherapy, n (%)	13.0%	4.3%	17.4%
Yes	6 (13.0)	2 (4.3)	8 (17.4)
Received auto-HSCT therapy, n (%)	6.5%	6.5%	13%
Yes	3 (6.5)	3 (6.5)	6 (13.0)
Median follow-up time months, 95%CI	21.8 (15.6-28.0)	19.2 (17.4-21.0)	21.8 (16.5-27.1)

Data are n (%). ECOG, Eastern Cooperative Oncology Group; HSCT, Hematopoietic Stem Cells Transplantation.

### Efficacy and outcomes

A total of 46 patients were selected for efficacy evaluation. At the data cutoff point, the median follow-up time was 21.8 months (95% CI, 16.4–27.1), with none of the patients achieving a complete response (CR). By the data cutoff, 18 out of 46 patients (39.1%) had died. In the first-line treatment group, 5 out of 22 patients (22.7%) died before disease progression, while in the second-line treatment group, 13 out of 24 patients (54.1%) died. Disease progression was observed in 26 out of 46 patients (56.5%) treated with anlotinib and sintilimab; this consisted of 11 out of 22 patients (50.0%) in the first-line group and 15 out of 24 patients (62.5%) in the second-line group.

**Figure 1:**
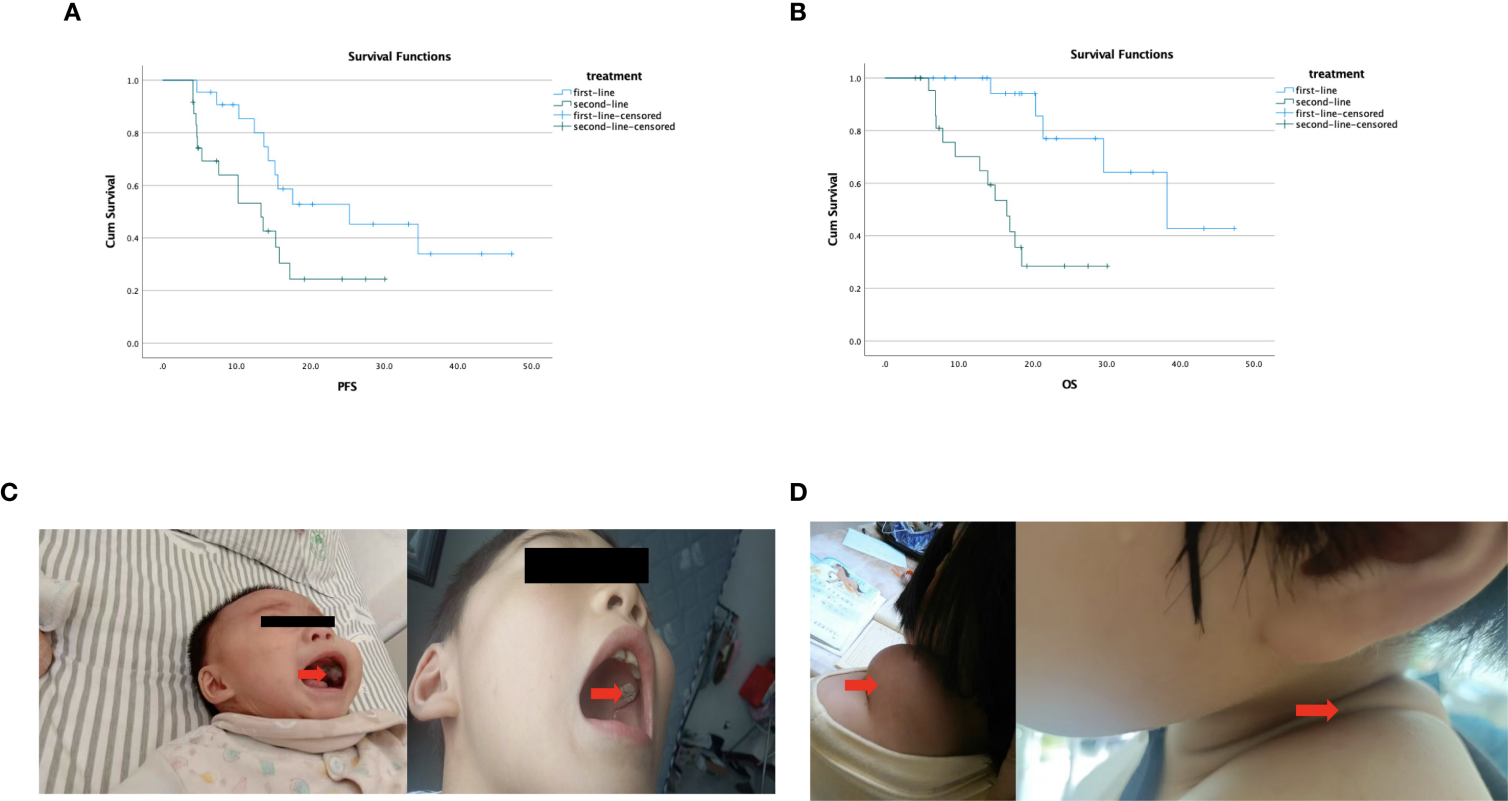
**(A)** Kaplan–Meier survival curve illustrating the differences in median PFS based on first- and second-line treatment; the difference in mPFS between groups reached statistical significance *(p* = 0.036). **(B)** Kaplan–Meier survival curve illustrating the differences in median OS based on first- and second-line treatment; there was a significant difference between the two groups (*p* < 0.001). **(C)** Patient 2 with infant pigmented neuroectodermal tumor—comparison of tumor size before and after maintenance treatment. **(D)** Patient 4 with desmoid fibromatosis—after 1 year of anlotinib combined with sintilimab maintenance treatment, the tumor was significantly reduced.

In the first-line treatment group, 3/22 (13.6%) patients had PR and 8/22 (36.4%) patients had SD. In the second-line group, 2/24 (8.3%) patients and 7/24 (29.2%) patients had PR and SD, respectively. As shown in **Table 2**, the mPFS was 25.3 months (95% CI, 7.0–43.6) in the first-line treatment and 13.3 months (95% CI, 7.2–19.4) in the second-line treatment; the difference in mPFS between groups reached statistical significance (*p* = 0.036). The mOS in the first-line and second-line treatment groups was 38.2 months (95% CI, 22.2–54.1) and 16.5 months (95% CI, 12.6–20.4), respectively (*p* < 0.001). The DCR was 50.0% (11/22; 95% CI, 28–72) and 37.5% (9/24; 95% CI, 19–59) in the first-line and second-line groups, respectively. There were no significant differences in DCR (*p* = 0.393) between the two groups (**Figure 1**). Among them, patient 2 and patient 4 exhibited the most favorable tumor responses, as demonstrated by PR. Patient 2, with infant pigmented neuroectodermal tumor, first developed the disease at the age of 6 months. After the first surgery, he was given alternating treatment with OPEC (VCR 1.5 mg/m^2^ intravenous injection on day 1, CTX 1.2 g/m^2^ intravenous injection on day 1, DDP 90 mg/m^2^ intravenous injection on day 2, VP-16–150 mg/m^2^ intravenous injection on day 4) and OPAC (VCR 1.5 mg/m^2^ intravenous injection on day 1, CTX 1.2 g/m^2^ intravenous injection on day 1, DDP 90 mg/m^2^ intravenous injection on day 2, ADR 30 mg/m^2^ intravenous injection on day 4) regimens without maintenance treatment. The tumor relapsed 3 months later. After the second surgery, he was given 6 cycles of dacarbazine combined with vindesine, and the tumor was smaller than before. He began to receive anlotinib combined with sintilimab maintenance treatment in November 2019. He has achieved partial remission and his condition remains stable, with a PFS of 47.3 months (**Figure 1C**). Patient 4, with desmoid fibromatosis and thoracic metastasis, received 8 cycles of liposomal doxorubicin chemotherapy (30 mg/m²), followed by 1 year of anlotinib combined with sintilimab maintenance therapy starting in February 2020. Maintenance therapy was discontinued due to rash and foot pain. At follow-up, the neck and chest masses gradually shrank and remained stable, with a PFS of 43.2 months (**Figure 1D**).

**Table 2:** Treatment responses.

Clinical Evaluation	First-line treatment	Second-line treatment	*P* value
mPFS (month,95%CI)	25.3 (7.03-43.6)	13.3 (7.2-19.4)	0.036
mOS (month,95%CI)	38.2 (22.2-54.2)	16.5 (12.6-20.4)	0.003
mFu	21.8 (15.63-27.97)	19.2 (17.44-20.96)	0.226
DCR (%,95%CI)	50.0 (28-72)	37.5 (19-59)	0.393
IQR	16.3-26.3	14.3-27.5	

This means the *p* value is less than 0.05, which indicates statistically significant.

### Safety outcomes

The majority of adverse events related to maintenance treatment with anlotinib combined with sintilimab were mild or moderate in severity. As shown in **Table 3**, the incidence of adverse events was 76% (35/46); vomiting (8.7%), fatigue (10.9%), and nausea (8.7%) were the most common adverse events. The incidence of grade 3 or 4 adverse events was 13.0% (6/46), which included both non-hematological and hematological AEs. Among the non-hematological AEs, grade 3 hypertension (n = 1), grade 3 abdominal pain (n = 1), and grade 3 hand–foot syndrome (n = 1) were observed. Among the patients who developed grade 3–4 hematological adverse events, one patient had received HSCT, and the remaining patients had received radiotherapy. This suggests that anemia and thrombocytopenia may be associated with high-dose chemotherapy and radiation therapy.

**Table 3:** Possible treatment-related adverse events.

AEs	Any grade	Grade 1-2	Grade 3-4
Non-hematological AEs	28 (60.9%)	25 (54.3%)	3 (6.5%)
Fatigue	5 (10.9%)	5 (10.9%)	0
Vomiting	4 (8.7%)	4 (8.7%)	0
Hypertension	3 (6.5%)	2 (4.3%)	1 (2.2%)
Headache	2 (4.3%)	2 (4.3%)	0
Nausea	4 (8.7%)	4 (8.7%)	0
Rash, maculopapular	1 (2.2%)	1 (2.2%)	0
Aspartate aminotransferase increased	2 (4.3%)	2 (4.3%)	0
Alanine aminotransferase increased	2 (4.3%)	2 (4.3%)	0
Abdominal pain	2 (4.3%)	1 (2.2%)	1 (2.2%)
Hand foot syndrome	2 (4.3%)	1 (2.2%)	1 (2.2%)
Pruritus	1 (2.2%)	1 (2.2%)	0
Hematological AEs	7 (15.2%)	4 (8.7%)	3 (6.5%)
Neutropenia	3 (6.5%)	3 (6.5%)	0
Thrombocytopenia	1 (2.2%)	0	1 (2.2%)
Anemia	3 (6.5%)	1 (2.2%)	2 (4.3%)

The primary treatment-related adverse events included hypertension (n=3, 6.5%), fatigue (n=5, 10.9%), nausea (n=4, 8.7%), and others. Grade 3–4 adverse events included anemia (n = 2, 4.3%), hypertension (n = 1, 2.2%), abdominal pain (n = 1, 2.2%), and hand–foot syndrome (n = 1, 2.2%).

At the end of follow-up, dose reduction of anlotinib occurred in 5 patients (14%), with the dose reduced by 20% per day. For patients with grade 3–4 adverse events, anlotinib was temporarily discontinued until the adverse events resolved to grade ≤1 (median duration of discontinuation: 5 days; range, 3–8 days). Treatment was then resumed at a reduced dose (from the initial 12 mg/day to 8 mg/day). None of these patients required further dose reduction or discontinuation, and all completed the planned course of treatment. One patient (2.2%) discontinued anlotinib treatment because of hypertension. No treatment-related deaths occurred.

## Discussion

Advanced pediatric tumors are prone to recurrence even if complete remission or stable disease is achieved (27). The dismal prognosis highlights the importance of developing novel therapies. Compared with the booming adult oncology drug market and research and development, the pediatric oncology drug market seems deserted (28, 29). Therefore, it is common to use drugs off-label in the treatment of childhood tumors (30). Compared with the existing published literature, this study is the first evaluation of the efficacy and safety of maintenance therapy using anlotinib alongside sintilimab for first-line and second-line treatment of advanced pediatric tumors. In addition, we are the first to evaluate the impact of maintenance therapy after first-line treatment and maintenance therapy after second-line treatment on patient survival.

As presented above, the results from our research center demonstrated that maintenance therapy initiated after first-line treatment without tumor progression yielded superior outcomes compared to maintenance therapy administered following second-line treatment, which was initiated after poor response to first-line therapy and subsequent tumor stabilization. These findings indicate that the efficacy of maintenance treatment in pediatric oncology is associated with the tumor’s prior response to chemotherapy, with significantly better outcomes observed when maintenance therapy is initiated after first-line treatment rather than second-line treatment. Early initiation of anlotinib combined with sintilimab maintenance therapy exhibited promising efficacy and favorable tolerability in pediatric cancer patients. The observed therapeutic synergy between anlotinib and sintilimab may reflect anlotinib’s immunomodulatory properties. Preclinical studies demonstrate that anlotinib normalizes tumor vasculature, reduces VEGF-mediated immunosuppression, and enhances CD8+ T-cell infiltration (31). Additionally, anlotinib has been shown to potentiate PD-1 blockade efficacy by downregulating STAT3-driven PD-L1 expression in sarcoma models (32). These mechanisms align with our clinical findings of prolonged PFS (25.3 months in first-line) and support the dual targeting of angiogenesis and immune checkpoints in pediatric tumors. Future studies should explore biomarker-driven patient selection (e.g., PD-L1/VEGF status) to refine this strategy.

In a real-world study by Yang (33), the combination of anti-angiogenic drugs and immunotherapy (anti-PD-1/PD-L1) demonstrated promising antitumor activity and tolerability in patients with extensive-stage small cell lung cancer (ES-SCLC). The inclusion of anlotinib in the maintenance therapy did not increase the toxicity of ICI monotherapy and maintained a manageable safety profile. In a retrospective study of advanced biliary tract cancer, anlotinib combined with anti-PD-1 as second-line treatment in 78 patients yielded a DCR of 87.2% and a PFS of 7.9 months (95% CI, 4.35–11.45), demonstrating significantly better outcomes compared to the chemotherapy group (34). Compared with their results, the DCR in our study was lower, which may be due to differences in immunobiological subtypes or PD-1 expression levels of solid tumors in children included in our study versus those in adults. These differences are similar to the clinical trial results reported by Kara et al. (ADVL1412) (35).

In addition, compared with the analysis results from the Affiliated Cancer Hospital of Sun Yat-sen University (NCT04400851) (15), the proportion of patients with grade 3 or higher treatment-related adverse reactions of sintilimab at different doses of 1, 3, and 10 mg/kg was 10%, and the adverse reaction rate in this study was 13%, which is similar to the results of the above clinical trials. This shows that the addition of anlotinib did not increase the incidence of serious adverse drug reactions.

In this study, there were several reasons for choosing anlotinib combined with sintilimab for maintenance treatment in advanced pediatric cancer. First, anti-angiogenic therapy can modulate the tumor immunosuppressive microenvironment and eliminate immunosuppression; therefore, the combination of anti-angiogenic drugs and immunotherapy holds promise in overcoming primary and secondary immune resistance (36). Secondly, anlotinib and sintilimab are both drugs independently developed in China, and they are relatively affordable. As maintenance treatment, they are more acceptable to patients with limited financial means. Third, these two drugs have a low incidence of hematological toxicity, making them easier to tolerate in patients with damaged bone marrow who have previously received high-dose chemotherapy, radiotherapy, or hematopoietic stem cell transplantation (37).

The present study faced various limitations. Our study has significant limitations, primarily including a small sample size (n=46) and its retrospective nature. The retrospective design inherently carries a risk of selection bias, as patient enrollment depends on the availability of complete medical records and follow-up data—this may lead to the exclusion of patients with more rapid disease progression, which in turn could overstate the observed treatment effects. Additionally, the small sample size limits our ability to detect subtle yet clinically meaningful differences, particularly in subgroup analyses stratified by tumor type. Future prospective, multicenter trials involving larger and more representative cohorts are necessary to validate our findings and reduce these biases.

## Conclusion

In conclusion, this study describes childhood tumor patients who benefited from anlotinib plus sintilimab maintenance therapy following first- or second-line chemotherapy-based treatment. The maintenance therapy represents a potentially reliable and beneficial option for patients who have achieved an objective response or stable disease (SD) after undergoing chemotherapy-based treatment for advanced pediatric tumors.

## Data Availability

The original contributions presented in the study are included in the article/supplementary material. Further inquiries can be directed to the corresponding author.
